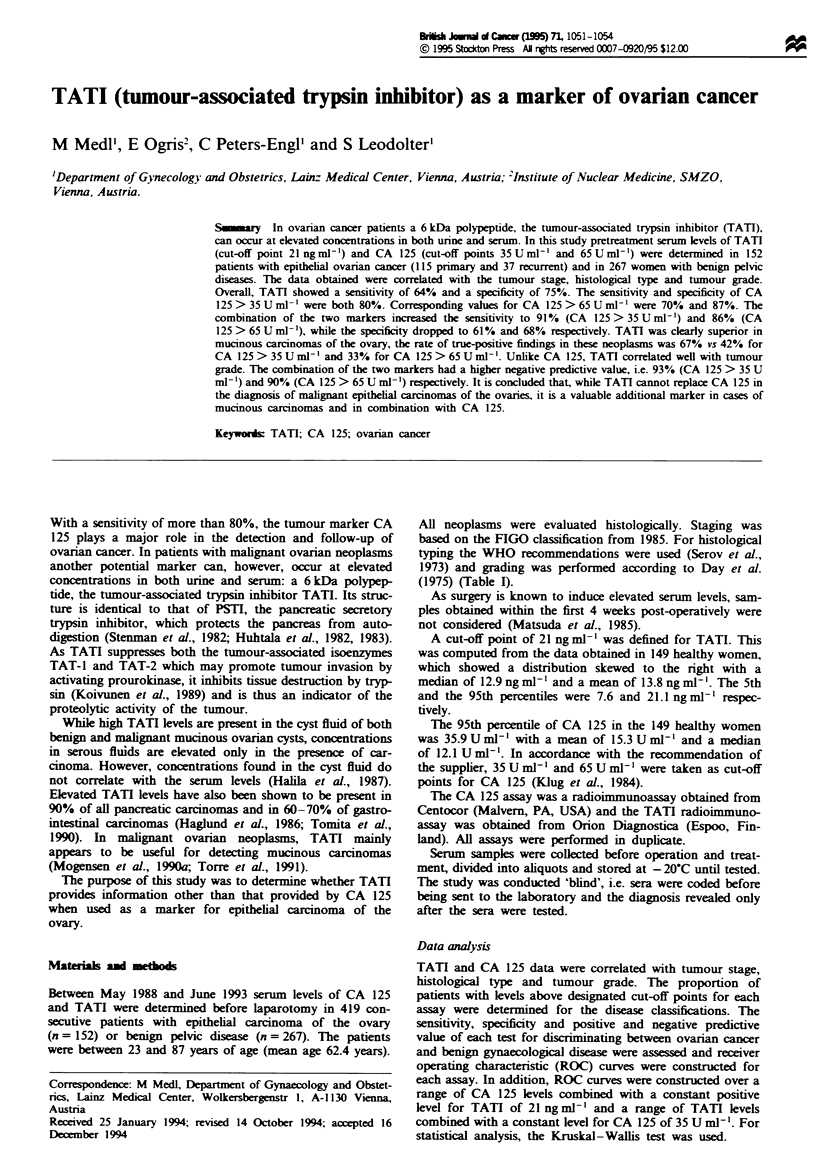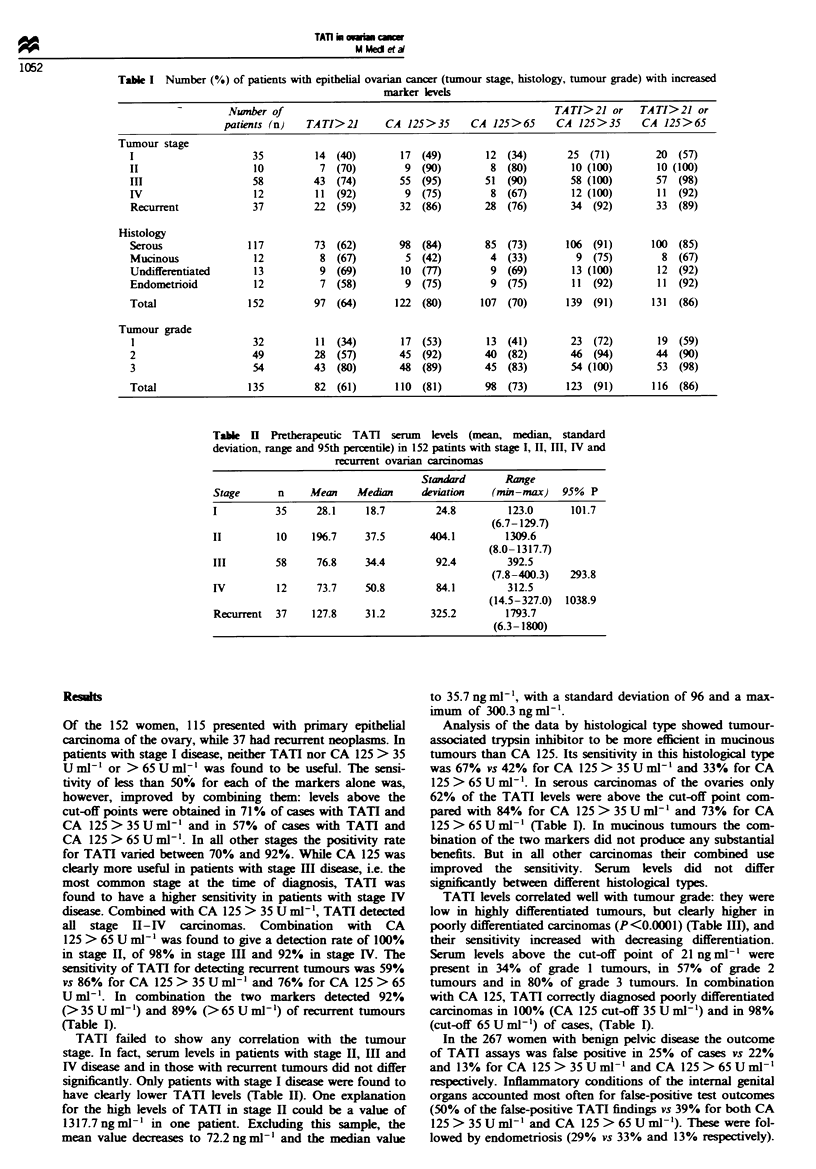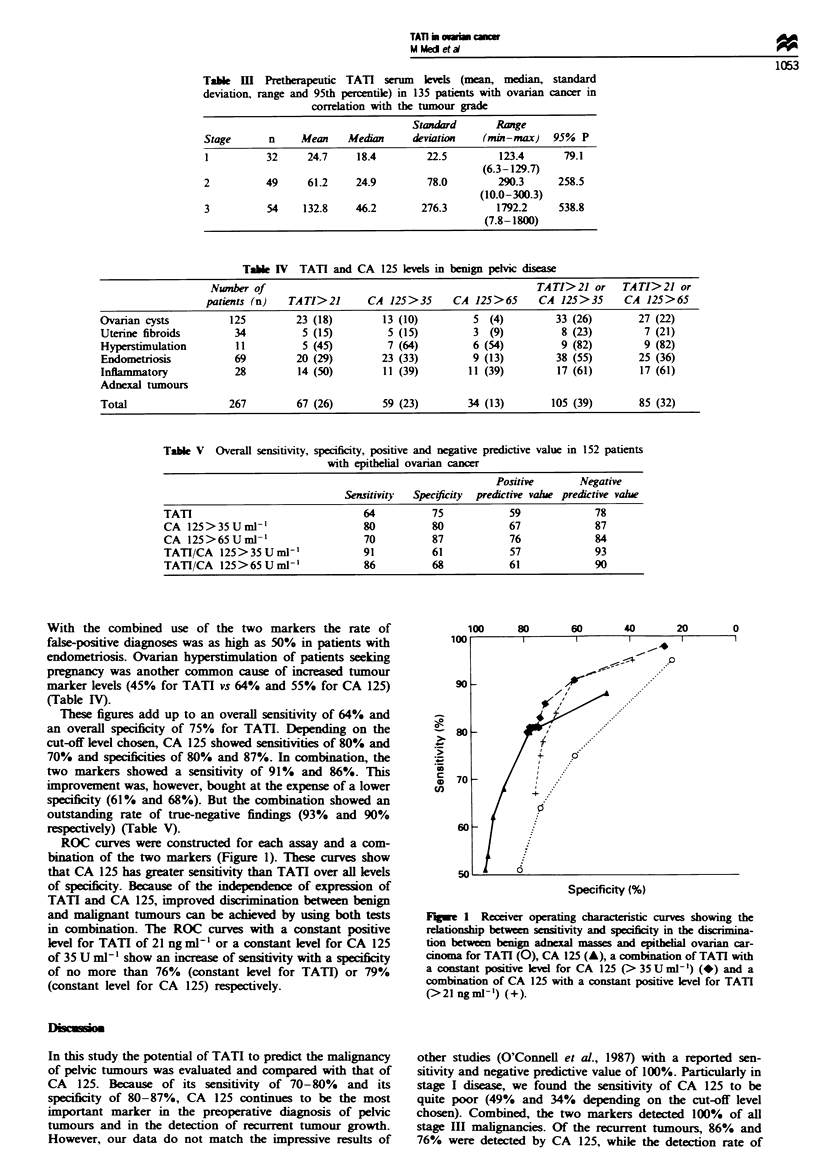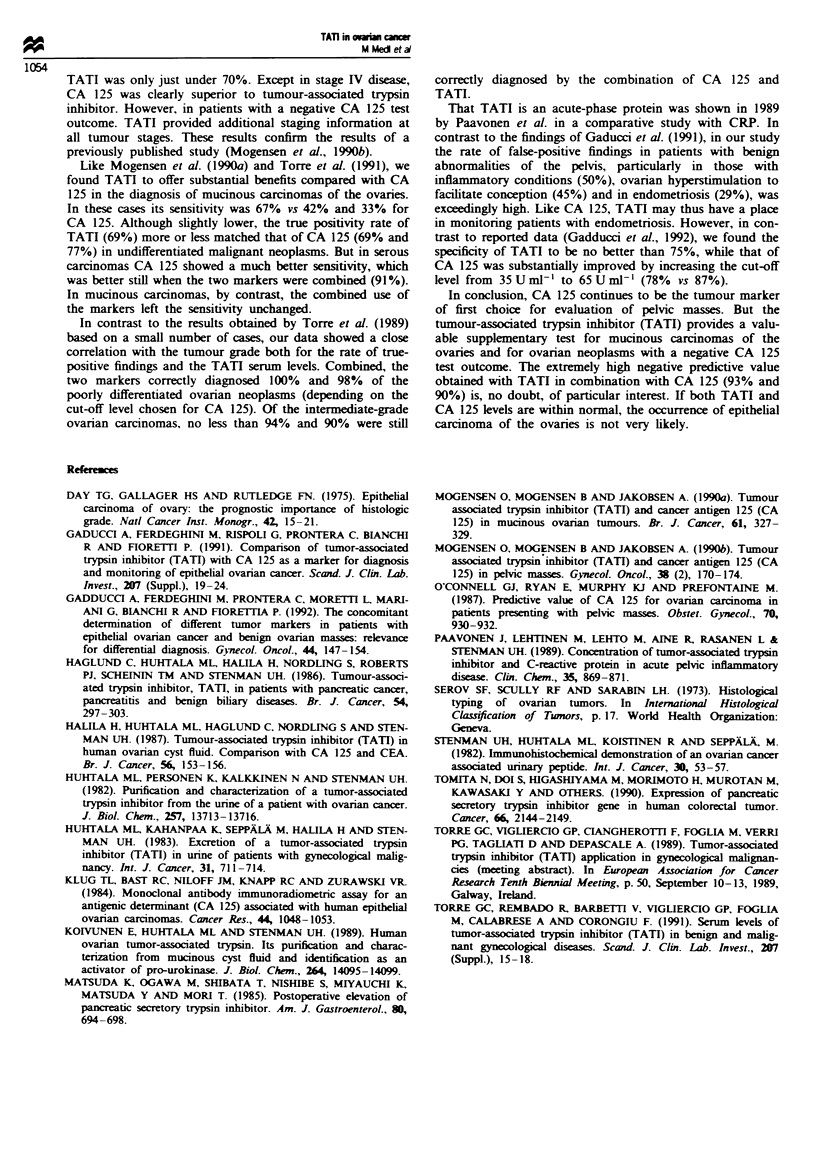# TATI (tumour-associated trypsin inhibitor) as a marker of ovarian cancer.

**DOI:** 10.1038/bjc.1995.202

**Published:** 1995-05

**Authors:** M. Medl, E. Ogris, C. Peters-Engl, S. Leodolter

**Affiliations:** Department of Gynecology and Obstetrics, Lainz Medical Center, Vienna, Austria.

## Abstract

In ovarian cancer patients a 6 kDa polypeptide, the tumour-associated trypsin inhibitor (TATI), can occur at elevated concentrations in both urine and serum. In this study pretreatment serum levels of TATI (cut-off point 21 ng ml-1) and CA 125 (cut-off points 35 U ml-1 and 65 U ml-1) were determined in 152 patients with epithelial ovarian cancer (115 primary and 37 recurrent) and in 267 women with benign pelvic diseases. The data obtained were correlated with the tumor stage, histological type and tumour grade. Overall, TATI showed a sensitivity of 64% and a specificity of 75%. The sensitivity and specificity of CA 125 > 35 U ml-1 were both 80%. Corresponding values for CA 125 > 65 U ml-1 were 70% and 87%. The combination of the two markers increased the sensitivity to 91% (CA 125 > 35 U ml-1) and 86% (CA 125 > 65 U ml-1), while the specificity dropped to 61% and 68% respectively. TATI was clearly superior in mucinous carcinomas of the ovary, the rate of true-positive findings in these neoplasms was 67% vs 42% for CA 125 > 35 U ml-1 and 33% for CA 125 > 65 U ml-1. Unlike CA 125, TATI correlated well with tumour grade. The combination of the two markers had a higher negative predictive value, i.e. 93% (CA 125 > 35 U ml-1) and 90% (CA 125 > 65 U ml-1) respectively. It is concluded that, while TATI cannot replace CA 125 in the diagnosis of malignant epithelial carcinomas of the ovaries, it is a valuable additional marker in cases of mucinous carcinomas and in combination with CA 125.


British Journal of Cancer (1995) 71, 1051-1054

© 1995 Stockton Press All rights reserved 0007-0920/95 $12.00

### TATI (tumour-associated trypsin inhibitor) as a marker of ovarian cancer

M Medl[1], E Ogris[2], C Peters-Engl[1] and S Leodolter[1]

[1]*Department of Gynecology and Obstetrics, Lainz Medical Center, Vienna, Austria;* [2]*Institute of Nuclear Medicine, SMZO, Vienna, Austria.*

**Summary** In ovarian cancer patients a 6 kDa polypeptide, the tumour-associated trypsin inhibitor (TATI), can occur at elevated concentrations in both urine and serum. In this study pretreatment serum levels of TATI (cut-off point 21 ng ml$^{-1}$) and CA 125 (cut-off points 35 U ml$^{-1}$ and 65 U ml$^{-1}$) were determined in 152 patients with epithelial ovarian cancer (115 primary and 37 recurrent) and in 267 women with benign pelvic diseases. The data obtained were correlated with the tumour stage, histological type and tumour grade. Overall, TATI showed a sensitivity of 64% and a specificity of 75%. The sensitivity and specificity of CA 125 > 35 U ml$^{-1}$ were both 80%. Corresponding values for CA 125 > 65 U ml$^{-1}$ were 70% and 87%. The combination of the two markers increased the sensitivity to 91% (CA 125 > 35 U ml$^{-1}$) and 86% (CA 125 > 65 U ml$^{-1}$), while the specificity dropped to 61% and 68% respectively. TATI was clearly superior in mucinous carcinomas of the ovary, the rate of true-positive findings in these neoplasms was 67% *vs* 42% for CA 125 > 35 U ml$^{-1}$ and 33% for CA 125 > 65 U ml$^{-1}$. Unlike CA 125, TATI correlated well with tumour grade. The combination of the two markers had a higher negative predictive value, i.e. 93% (CA 125 > 35 U ml$^{-1}$) and 90% (CA 125 > 65 U ml$^{-1}$) respectively. It is concluded that, while TATI cannot replace CA 125 in the diagnosis of malignant epithelial carcinomas of the ovaries, it is a valuable additional marker in cases of mucinous carcinomas and in combination with CA 125.

**Keywords:** TATI; CA 125; ovarian cancer

With a sensitivity of more than 80%, the tumour marker CA 125 plays a major role in the detection and follow-up of ovarian cancer. In patients with malignant ovarian neoplasms another potential marker can, however, occur at elevated concentrations in both urine and serum: a 6 kDa polypeptide, the tumour-associated trypsin inhibitor TATI. Its structure is identical to that of PSTI, the pancreatic secretory trypsin inhibitor, which protects the pancreas from autodigestion (Stenman *et al.*, 1982; Huhtala *et al.*, 1982, 1983). As TATI suppresses both the tumour-associated isoenzymes TAT-1 and TAT-2 which may promote tumour invasion by activating prourokinase, it inhibits tissue destruction by trypsin (Koivunen *et al.*, 1989) and is thus an indicator of the proteolytic activity of the tumour.

While high TATI levels are present in the cyst fluid of both benign and malignant mucinous ovarian cysts, concentrations in serous fluids are elevated only in the presence of carcinoma. However, concentrations found in the cyst fluid do not correlate with the serum levels (Halila *et al.*, 1987). Elevated TATI levels have also been shown to be present in 90% of all pancreatic carcinomas and in 60-70% of gastrointestinal carcinomas (Haglund *et al.*, 1986; Tomita *et al.*, 1990). In malignant ovarian neoplasms, TATI mainly appears to be useful for detecting mucinous carcinomas (Mogensen *et al.*, 1990*a*; Torre *et al.*, 1991).

The purpose of this study was to determine whether TATI provides information other than that provided by CA 125 when used as a marker for epithelial carcinoma of the ovary.

### Materials and methods

Between May 1988 and June 1993 serum levels of CA 125 and TATI were determined before laparotomy in 419 consecutive patients with epithelial carcinoma of the ovary ($n = 152$) or benign pelvic disease ($n = 267$). The patients were between 23 and 87 years of age (mean age 62.4 years). All neoplasms were evaluated histologically. Staging was based on the FIGO classification from 1985. For histological typing the WHO recommendations were used (Serov *et al.*, 1973) and grading was performed according to Day *et al.* (1975) (Table I).

As surgery is known to induce elevated serum levels, samples obtained within the first 4 weeks post-operatively were not considered (Matsuda *et al.*, 1985).

A cut-off point of 21 ng ml$^{-1}$ was defined for TATI. This was computed from the data obtained in 149 healthy women, which showed a distribution skewed to the right with a median of 12.9 ng ml$^{-1}$ and a mean of 13.8 ng ml$^{-1}$. The 5th and the 95th percentiles were 7.6 and 21.1 ng ml$^{-1}$ respectively.

The 95th percentile of CA 125 in the 149 healthy women was 35.9 U ml$^{-1}$ with a mean of 15.3 U ml$^{-1}$ and a median of 12.1 U ml$^{-1}$. In accordance with the recommendation of the supplier, 35 U ml$^{-1}$ and 65 U ml$^{-1}$ were taken as cut-off points for CA 125 (Klug *et al.*, 1984).

The CA 125 assay was a radioimmunoassay obtained from Centocor (Malvern, PA, USA) and the TATI radioimmunoassay was obtained from Orion Diagnostica (Espoo, Finland). All assays were performed in duplicate.

Serum samples were collected before operation and treatment, divided into aliquots and stored at −20°C until tested. The study was conducted 'blind', i.e. sera were coded before being sent to the laboratory and the diagnosis revealed only after the sera were tested.

### Data analysis

TATI and CA 125 data were correlated with tumour stage, histological type and tumour grade. The proportion of patients with levels above designated cut-off points for each assay were determined for the disease classifications. The sensitivity, specificity and positive and negative predictive value of each test for discriminating between ovarian cancer and benign gynaecological disease were assessed and receiver operating characteristic (ROC) curves were constructed for each assay. In addition, ROC curves were constructed over a range of CA 125 levels combined with a constant positive level for TATI of 21 ng ml$^{-1}$ and a range of TATI levels combined with a constant level for CA 125 of 35 U ml$^{-1}$. For statistical analysis, the Kruskal-Wallis test was used.

Correspondence: M Medl, Department of Gynaecology and Obstetrics, Lainz Medical Center, Wolkersbergenstr 1, A-1130 Vienna, Austria
Received 25 January 1994; revised 14 October 1994; accepted 16 December 1994

**Table I** Number (%) of patients with epithelial ovarian cancer (tumour stage, histology, tumour grade) with increased marker levels

| | *Number of patients (n)* | *TATI>21* | *CA 125>35* | *CA 125>65* | *TATI>21 or CA 125>35* | *TATI>21 or CA 125>65* |
|---|---|---|---|---|---|---|
| Tumour stage | | | | | | |
| I | 35 | 14 (40) | 17 (49) | 12 (34) | 25 (71) | 20 (57) |
| II | 10 | 7 (70) | 9 (90) | 8 (80) | 10 (100) | 10 (100) |
| III | 58 | 43 (74) | 55 (95) | 51 (90) | 58 (100) | 57 (98) |
| IV | 12 | 11 (92) | 9 (75) | 8 (67) | 12 (100) | 11 (92) |
| Recurrent | 37 | 22 (59) | 32 (86) | 28 (76) | 34 (92) | 33 (89) |
| Histology | | | | | | |
| Serous | 117 | 73 (62) | 98 (84) | 85 (73) | 106 (91) | 100 (85) |
| Mucinous | 12 | 8 (67) | 5 (42) | 4 (33) | 9 (75) | 8 (67) |
| Undifferentiated | 13 | 9 (69) | 10 (77) | 9 (69) | 13 (100) | 12 (92) |
| Endometrioid | 12 | 7 (58) | 9 (75) | 9 (75) | 11 (92) | 11 (92) |
| Total | 152 | 97 (64) | 122 (80) | 107 (70) | 139 (91) | 131 (86) |
| Tumour grade | | | | | | |
| 1 | 32 | 11 (34) | 17 (53) | 13 (41) | 23 (72) | 19 (59) |
| 2 | 49 | 28 (57) | 45 (92) | 40 (82) | 46 (94) | 44 (90) |
| 3 | 54 | 43 (80) | 48 (89) | 45 (83) | 54 (100) | 53 (98) |
| Total | 135 | 82 (61) | 110 (81) | 98 (73) | 123 (91) | 116 (86) |

**Table II** Pretherapeutic TATI serum levels (mean, median, standard deviation, range and 95th percentile) in 152 patints with stage I, II, III, IV and recurrent ovarian carcinomas

| *Stage* | n | *Mean* | *Median* | *Standard deviation* | *Range (min-max)* | *95% P* |
|---|---|---|---|---|---|---|
| I | 35 | 28.1 | 18.7 | 24.8 | 123.0 (6.7-129.7) | 101.7 |
| II | 10 | 196.7 | 37.5 | 404.1 | 1309.6 (8.0-1317.7) | |
| III | 58 | 76.8 | 34.4 | 92.4 | 392.5 (7.8-400.3) | 293.8 |
| IV | 12 | 73.7 | 50.8 | 84.1 | 312.5 (14.5-327.0) | 1038.9 |
| Recurrent | 37 | 127.8 | 31.2 | 325.2 | 1793.7 (6.3-1800) | |

### Results

Of the 152 women, 115 presented with primary epithelial carcinoma of the ovary, while 37 had recurrent neoplasms. In patients with stage I disease, neither TATI nor CA 125 > 35 U ml$^{-1}$ or > 65 U ml$^{-1}$ was found to be useful. The sensitivity of less than 50% for each of the markers alone was, however, improved by combining them: levels above the cut-off points were obtained in 71% of cases with TATI and CA 125 > 35 U ml$^{-1}$ and in 57% of cases with TATI and CA 125 > 65 U ml$^{-1}$. In all other stages the positivity rate for TATI varied between 70% and 92%. While CA 125 was clearly more useful in patients with stage III disease, i.e. the most common stage at the time of diagnosis, TATI was found to have a higher sensitivity in patients with stage IV disease. Combined with CA 125 > 35 U ml$^{-1}$, TATI detected all stage II-IV carcinomas. Combination with CA 125 > 65 U ml$^{-1}$ was found to give a detection rate of 100% in stage II, of 98% in stage III and 92% in stage IV. The sensitivity of TATI for detecting recurrent tumours was 59% *vs* 86% for CA 125 > 35 U ml$^{-1}$ and 76% for CA 125 > 65 U ml$^{-1}$. In combination the two markers detected 92% (> 35 U ml$^{-1}$) and 89% (> 65 U ml$^{-1}$) of recurrent tumours (Table I).

TATI failed to show any correlation with the tumour stage. In fact, serum levels in patients with stage II, III and IV disease and in those with recurrent tumours did not differ significantly. Only patients with stage I disease were found to have clearly lower TATI levels (Table II). One explanation for the high levels of TATI in stage II could be a value of 1317.7 ng ml$^{-1}$ in one patient. Excluding this sample, the mean value decreases to 72.2 ng ml$^{-1}$ and the median value to 35.7 ng ml$^{-1}$, with a standard deviation of 96 and a maximum of 300.3 ng ml$^{-1}$.

Analysis of the data by histological type showed tumour-associated trypsin inhibitor to be more efficient in mucinous tumours than CA 125. Its sensitivity in this histological type was 67% *vs* 42% for CA 125 > 35 U ml$^{-1}$ and 33% for CA 125 > 65 U ml$^{-1}$. In serous carcinomas of the ovaries only 62% of the TATI levels were above the cut-off point compared with 84% for CA 125 > 35 U ml$^{-1}$ and 73% for CA 125 > 65 U ml$^{-1}$ (Table I). In mucinous tumours the combination of the two markers did not produce any substantial benefits. But in all other carcinomas their combined use improved the sensitivity. Serum levels did not differ significantly between different histological types.

TATI levels correlated well with tumour grade: they were low in highly differentiated tumours, but clearly higher in poorly differentiated carcinomas ($P$<0.0001) (Table III), and their sensitivity increased with decreasing differentiation. Serum levels above the cut-off point of 21 ng ml$^{-1}$ were present in 34% of grade 1 tumours, in 57% of grade 2 tumours and in 80% of grade 3 tumours. In combination with CA 125, TATI correctly diagnosed poorly differentiated carcinomas in 100% (CA 125 cut-off 35 U ml$^{-1}$) and in 98% (cut-off 65 U ml$^{-1}$) of cases, (Table I).

In the 267 women with benign pelvic disease the outcome of TATI assays was false positive in 25% of cases *vs* 22% and 13% for CA 125 > 35 U ml$^{-1}$ and CA 125 > 65 U ml$^{-1}$ respectively. Inflammatory conditions of the internal genital organs accounted most often for false-positive test outcomes (50% of the false-positive TATI findings *vs* 39% for both CA 125 > 35 U ml$^{-1}$ and CA 125 > 65 U ml$^{-1}$). These were followed by endometriosis (29% *vs* 33% and 13% respectively).

**Table III** Pretherapeutic TATI serum levels (mean, median, standard deviation, range and 95th percentile) in 135 patients with ovarian cancer in correlation with the tumour grade

| *Stage* | n | *Mean* | *Median* | *Standard deviation* | *Range (min–max)* | *95%* P |
|---|---|---|---|---|---|---|
| 1 | 32 | 24.7 | 18.4 | 22.5 | 123.4 (6.3–129.7) | 79.1 |
| 2 | 49 | 61.2 | 24.9 | 78.0 | 290.3 (10.0–300.3) | 258.5 |
| 3 | 54 | 132.8 | 46.2 | 276.3 | 1792.2 (7.8–1800) | 538.8 |

**Table IV** TATI and CA 125 levels in benign pelvic disease

| | *Number of patients (n)* | *TATI>21* | *CA 125>35* | *CA 125>65* | *TATI>21 or CA 125>35* | *TATI>21 or CA 125>65* |
|---|---|---|---|---|---|---|
| Ovarian cysts | 125 | 23 (18) | 13 (10) | 5 (4) | 33 (26) | 27 (22) |
| Uterine fibroids | 34 | 5 (15) | 5 (15) | 3 (9) | 8 (23) | 7 (21) |
| Hyperstimulation | 11 | 5 (45) | 7 (64) | 6 (54) | 9 (82) | 9 (82) |
| Endometriosis | 69 | 20 (29) | 23 (33) | 9 (13) | 38 (55) | 25 (36) |
| Inflammatory Adnexal tumours | 28 | 14 (50) | 11 (39) | 11 (39) | 17 (61) | 17 (61) |
| Total | 267 | 67 (26) | 59 (23) | 34 (13) | 105 (39) | 85 (32) |

**Table V** Overall sensitivity, specificity, positive and negative predictive value in 152 patients with epithelial ovarian cancer

| | *Sensitivity* | *Specificity* | *Positive predictive value* | *Negative predictive value* |
|---|---|---|---|---|
| TATI | 64 | 75 | 59 | 78 |
| CA 125>35 U ml$^{-1}$ | 80 | 80 | 67 | 87 |
| CA 125>65 U ml$^{-1}$ | 70 | 87 | 76 | 84 |
| TATI/CA 125>35 U ml$^{-1}$ | 91 | 61 | 57 | 93 |
| TATI/CA 125>65 U ml$^{-1}$ | 86 | 68 | 61 | 90 |

With the combined use of the two markers the rate of false-positive diagnoses was as high as 50% in patients with endometriosis. Ovarian hyperstimulation of patients seeking pregnancy was another common cause of increased tumour marker levels (45% for TATI *vs* 64% and 55% for CA 125) (Table IV).

These figures add up to an overall sensitivity of 64% and an overall specificity of 75% for TATI. Depending on the cut-off level chosen, CA 125 showed sensitivities of 80% and 70% and specificities of 80% and 87%. In combination, the two markers showed a sensitivity of 91% and 86%. This improvement was, however, bought at the expense of a lower specificity (61% and 68%). But the combination showed an outstanding rate of true-negative findings (93% and 90% respectively) (Table V).

ROC curves were constructed for each assay and a combination of the two markers (Figure 1). These curves show that CA 125 has greater sensitivity than TATI over all levels of specificity. Because of the independence of expression of TATI and CA 125, improved discrimination between benign and malignant tumours can be achieved by using both tests in combination. The ROC curves with a constant positive level for TATI of 21 ng ml$^{-1}$ or a constant level for CA 125 of 35 U ml$^{-1}$ show an increase of sensitivity with a specificity of no more than 76% (constant level for TATI) or 79% (constant level for CA 125) respectively.

**Figure 1** Receiver operating characteristic curves showing the relationship between sensitivity and specificity in the discrimination between benign adnexal masses and epithelial ovarian carcinoma for TATI (O), CA 125 (▲), a combination of TATI with a constant positive level for CA 125 (>35 U ml$^{-1}$) (◆) and a combination of CA 125 with a constant positive level for TATI (>21 ng ml$^{-1}$) (+).

### Discussion

In this study the potential of TATI to predict the malignancy of pelvic tumours was evaluated and compared with that of CA 125. Because of its sensitivity of 70–80% and its specificity of 80–87%, CA 125 continues to be the most important marker in the preoperative diagnosis of pelvic tumours and in the detection of recurrent tumour growth. However, our data do not match the impressive results of other studies (O'Connell *et al.*, 1987) with a reported sensitivity and negative predictive value of 100%. Particularly in stage I disease, we found the sensitivity of CA 125 to be quite poor (49% and 34% depending on the cut-off level chosen). Combined, the two markers detected 100% of all stage III malignancies. Of the recurrent tumours, 86% and 76% were detected by CA 125, while the detection rate of

TATI was only just under 70%. Except in stage IV disease, CA 125 was clearly superior to tumour-associated trypsin inhibitor. However, in patients with a negative CA 125 test outcome. TATI provided additional staging information at all tumour stages. These results confirm the results of a previously published study (Mogensen *et al.*, 1990*b*).

Like Mogensen *et al.* (1990*a*) and Torre *et al.* (1991), we found TATI to offer substantial benefits compared with CA 125 in the diagnosis of mucinous carcinomas of the ovaries. In these cases its sensitivity was 67% *vs* 42% and 33% for CA 125. Although slightly lower, the true positivity rate of TATI (69%) more or less matched that of CA 125 (69% and 77%) in undifferentiated malignant neoplasms. But in serous carcinomas CA 125 showed a much better sensitivity, which was better still when the two markers were combined (91%). In mucinous carcinomas, by contrast, the combined use of the markers left the sensitivity unchanged.

In contrast to the results obtained by Torre *et al.* (1989) based on a small number of cases, our data showed a close correlation with the tumour grade both for the rate of true-positive findings and the TATI serum levels. Combined, the two markers correctly diagnosed 100% and 98% of the poorly differentiated ovarian neoplasms (depending on the cut-off level chosen for CA 125). Of the intermediate-grade ovarian carcinomas, no less than 94% and 90% were still correctly diagnosed by the combination of CA 125 and TATI.

That TATI is an acute-phase protein was shown in 1989 by Paavonen *et al.* in a comparative study with CRP. In contrast to the findings of Gaducci *et al.* (1991), in our study the rate of false-positive findings in patients with benign abnormalities of the pelvis, particularly in those with inflammatory conditions (50%), ovarian hyperstimulation to facilitate conception (45%) and in endometriosis (29%), was exceedingly high. Like CA 125, TATI may thus have a place in monitoring patients with endometriosis. However, in contrast to reported data (Gadducci *et al.*, 1992), we found the specificity of TATI to be no better than 75%, while that of CA 125 was substantially improved by increasing the cut-off level from 35 U $ml^{-1}$ to 65 U $ml^{-1}$ (78% *vs* 87%).

In conclusion, CA 125 continues to be the tumour marker of first choice for evaluation of pelvic masses. But the tumour-associated trypsin inhibitor (TATI) provides a valuable supplementary test for mucinous carcinomas of the ovaries and for ovarian neoplasms with a negative CA 125 test outcome. The extremely high negative predictive value obtained with TATI in combination with CA 125 (93% and 90%) is, no doubt, of particular interest. If both TATI and CA 125 levels are within normal, the occurrence of epithelial carcinoma of the ovaries is not very likely.

### References

DAY TG, GALLAGER HS AND RUTLEDGE FN. (1975). Epithelial carcinoma of ovary: the prognostic importance of histologic grade. *Natl Cancer Inst. Monogr.*, **42**, 15–21.

GADUCCI A, FERDEGHINI M, RISPOLI G, PRONTERA C, BIANCHI R AND FIORETTI P. (1991). Comparison of tumor-associated trypsin inhibitor (TATI) with CA 125 as a marker for diagnosis and monitoring of epithelial ovarian cancer. *Scand. J. Clin. Lab. Invest.*, **207** (Suppl.), 19–24.

GADDUCCI A, FERDEGHINI M, PRONTERA C, MORETTI L, MARIANI G, BIANCHI R AND FIORETTIA P. (1992). The concomitant determination of different tumor markers in patients with epithelial ovarian cancer and benign ovarian masses: relevance for differential diagnosis. *Gynecol. Oncol.*, **44**, 147–154.

HAGLUND C, HUHTALA ML, HALILA H, NORDLING S, ROBERTS PJ, SCHEININ TM AND STENMAN UH. (1986). Tumour-associated trypsin inhibitor, TATI, in patients with pancreatic cancer, pancreatitis and benign biliary diseases. *Br. J. Cancer*, **54**, 297–303.

HALILA H, HUHTALA ML, HAGLUND C, NORDLING S AND STENMAN UH. (1987). Tumour-associated trypsin inhibitor (TATI) in human ovarian cyst fluid. Comparison with CA 125 and CEA. *Br. J. Cancer*, **56**, 153–156.

HUHTALA ML, PERSONEN K, KALKKINEN N AND STENMAN UH. (1982). Purification and characterization of a tumor-associated trypsin inhibitor from the urine of a patient with ovarian cancer. *J. Biol. Chem.*, **257**, 13713–13716.

HUHTALA ML, KAHANPAA K, SEPPÄLÄ M, HALILA H AND STENMAN UH. (1983). Excretion of a tumor-associated trypsin inhibitor (TATI) in urine of patients with gynecological malignancy. *Int. J. Cancer*, **31**, 711–714.

KLUG TL, BAST RC, NILOFF JM, KNAPP RC AND ZURAWSKI VR. (1984). Monoclonal antibody immunoradiometric assay for an antigenic determinant (CA 125) associated with human epithelial ovarian carcinomas. *Cancer Res.*, **44**, 1048–1053.

KOIVUNEN E, HUHTALA ML AND STENMAN UH. (1989). Human ovarian tumor-associated trypsin. Its purification and characterization from mucinous cyst fluid and identification as an activator of pro-urokinase. *J. Biol. Chem.*, **264**, 14095–14099.

MATSUDA K, OGAWA M, SHIBATA T, NISHIBE S, MIYAUCHI K, MATSUDA Y AND MORI T. (1985). Postoperative elevation of pancreatic secretory trypsin inhibitor. *Am. J. Gastroenterol.*, **80**, 694–698.

MOGENSEN O, MOGENSEN B AND JAKOBSEN A. (1990*a*). Tumour associated trypsin inhibitor (TATI) and cancer antigen 125 (CA 125) in mucinous ovarian tumours. *Br. J. Cancer*, **61**, 327–329.

MOGENSEN O, MOGENSEN B AND JAKOBSEN A. (1990*b*). Tumour associated trypsin inhibitor (TATI) and cancer antigen 125 (CA 125) in pelvic masses. *Gynecol. Oncol.*, **38** (2), 170–174.

O'CONNELL GJ, RYAN E, MURPHY KJ AND PREFONTAINE M. (1987). Predictive value of CA 125 for ovarian carcinoma in patients presenting with pelvic masses. *Obstet. Gynecol.*, **70**, 930–932.

PAAVONEN J, LEHTINEN M, LEHTO M, AINE R, RASANEN L & STENMAN UH. (1989). Concentration of tumor-associated trypsin inhibitor and C-reactive protein in acute pelvic inflammatory disease. *Clin. Chem.*, **35**, 869–871.

SEROV SF, SCULLY RF AND SARABIN LH. (1973). Histological typing of ovarian tumors. In *International Histological Classification of Tumors*, p. 17. World Health Organization: Geneva.

STENMAN UH, HUHTALA ML, KOISTINEN R AND SEPPÄLÄ M. (1982). Immunohistochemical demonstration of an ovarian cancer associated urinary peptide. *Int. J. Cancer*, **30**, 53–57.

TOMITA N, DOI S, HIGASHIYAMA M, MORIMOTO H, MUROTAN M, KAWASAKI Y AND OTHERS. (1990). Expression of pancreatic secretory trypsin inhibitor gene in human colorectal tumor. *Cancer*, **66**, 2144–2149.

TORRE GC, VIGLIERCIO GP, CIANGHEROTTI F, FOGLIA M, VERRI PG, TAGLIATI D AND DEPASCALE A. (1989). Tumor-associated trypsin inhibitor (TATI) application in gynecological malignancies (meeting abstract). In *European Association for Cancer Research Tenth Biennial Meeting*, p. 50, September 10–13, 1989, Galway, Ireland.

TORRE GC, REMBADO R, BARBETTI V, VIGLIERCIO GP, FOGLIA M, CALABRESE A AND CORONGIU F. (1991). Serum levels of tumor-associated trypsin inhibitor (TATI) in benign and malignant gynecological diseases. *Scand. J. Clin. Lab. Invest.*, **207** (Suppl.), 15–18.